# Current and Emerging Therapeutic Strategies for Limited- and Extensive-Stage Small-Cell Lung Cancer

**DOI:** 10.3390/medsci13030142

**Published:** 2025-08-18

**Authors:** Walid Shalata, Rashad Naamneh, Wenad Najjar, Mohnnad Asla, Adam Abu Gameh, Mahmoud Abu Amna, Leonard Saiegh, Abed Agbarya

**Affiliations:** 1 The Legacy Heritage Cancer Center, Larry Norton Institute, Soroka Medical Center, Beer-Sheva 8410501, Israel; 2 Goldman Medical School, Faculty of Health Sciences, Ben-Gurion University of the Negev, Beer-Sheva 8410501, Israel; 3 Neurosurgery Department, Soroka Medical Center, Beer-Sheva 8410501, Israel; 4 Neurosurgery Department, Hadassah Hebrew University Medical Center, Jerusalem 91200, Israel; 5 The Ruth and Bruce Rappaport Faculty of Medicine, Technion, Haifa 31096, Israel; mahmud_ab@clalit.org.il (M.A.A.);; 6 Institute of Endocrinology, Bnai Zion Medical Center, 47 Golomb St., Haifa 31048, Israel; 7 Oncology Department, Bnai Zion Medical Center, Haifa 3104701, Israel

**Keywords:** small-cell lung cancer, platinum agent and etoposide, prophylactic cranial irradiation, IMpower133 trial, CASPIAN trial, IMforte

## Abstract

Background: Small-cell lung cancer (SCLC) is a highly aggressive neuroendocrine malignancy characterized by rapid growth, early metastatic dissemination, and a dismal prognosis. For decades, treatment paradigms remained largely stagnant, particularly for extensive-stage disease (ES-SCLC). However, the last five years have witnessed a significant evolution in the therapeutic landscape. Methods: The information for this article was gathered by synthesizing data from several key sources. This article synthesizes the evidence supporting current standards of care for both limited-stage (LS-SCLC) and ES-SCLC, incorporating data from pivotal clinical trials, a network meta-analysis of first-line chemoimmunotherapy regimens, and a critical appraisal of international treatment guidelines, and a critical analysis of international treatment guidelines from prominent organizations like the National Comprehensive Cancer Network (NCCN) and the European Society for Medical Oncology (ESMO). This comprehensive approach allows for a robust and well-supported summary of the current therapeutic landscape. Results: For limited-stage SCLC (LS-SCLC), concurrent chemoradiotherapy (cCRT) remains the curative-intent standard, but its efficacy is now being augmented by consolidative immunotherapy, as demonstrated by the landmark ADRIATIC trial. The role of prophylactic cranial irradiation (PCI) in LS-SCLC is being re-evaluated in the era of high-sensitivity brain imaging and concerns over neurotoxicity. For ES-SCLC, the treatment paradigm has been fundamentally transformed by the integration of immune checkpoint inhibitors (ICIs) with platinum–etoposide chemotherapy, establishing a new standard of care that offers a modest but consistent survival benefit. Conclusions: The treatment of SCLC has been significantly advanced by the integration of immunotherapy, particularly for extensive-stage disease, which has established a new standard of care and improved patient outcomes. Looking to the future, the quest for predictive biomarkers and the development of novel therapeutic classes, such as Bi-specific T-cell Engagers (BiTEs) and antibody–drug conjugates, promise to build upon recent progress and offer new hope for improving the dismal prognosis associated with this disease.

## 1. Introduction

Lung cancer is the third most commonly diagnosed cancer in the United States and remains the leading cause of cancer-related deaths worldwide [1]. Small-cell lung cancer (SCLC), in particular, continues to pose a significant therapeutic challenge due to limited treatment options. SCLC represents approximately 10–20% of all lung malignancies and is distinguished by its exceptionally aggressive clinical behavior, including a rapid tumor volume doubling time, a high growth fraction, and a profound propensity for early, widespread metastatic dissemination [2,3]. Multiple studies have consistently documented the downward trajectory of SCLC incidence. For instance, data from the Surveillance, Epidemiology, and End Results (SEER) program in the United States shows a steady decrease in the age-adjusted incidence rate of SCLC. One analysis of the SEER database highlighted a drop from 8.8 cases per 100,000 people in the year 2000 to 4.8 per 100,000 in 2019 [4,5].

The etiology of SCLC is overwhelmingly linked to cigarette smoking, with carcinogens such as polycyclic aromatic hydrocarbons and N-nitrosamines driving its pathogenesis; only about 1% of cases are diagnosed in individuals who have never smoked [3,6]. This strong etiological association results in a high somatic tumor mutational burden, a characteristic feature of the disease.

The clinical presentation of SCLC is often dramatic and of rapid onset, with symptoms developing over just 8 to 12 weeks prior to diagnosis. Most tumors arise centrally in the major bronchi, leading to cough, dyspnea, and hemoptysis. Due to its rapid dissemination, the majority of patients—approximately two-thirds or 70%—present with extensive-stage disease (ES-SCLC) at the time of diagnosis, where the cancer has already spread beyond the confines of a single hemithorax. Common sites of metastasis include the contralateral lung, liver, adrenal glands, bone, and brain. This advanced stage at presentation precludes curative-intent therapy for most individuals and contributes to a grim overall prognosis, with a 5-year relative survival rate of less than 7%. While SCLC is initially highly sensitive to chemotherapy and radiation, acquired resistance develops almost universally and rapidly, leading to relapse and disease progression [6,7].

## 2. Pathobiology: From Histology to Molecular Subtypes

A sophisticated understanding of SCLC pathobiology is essential for interpreting its clinical behavior and developing rational therapeutic strategies.

### 2.1. Histopathology and Immunohistochemistry

Under light microscopy, SCLC is defined as a high-grade neuroendocrine tumor composed of primitive-appearing cells. The tumor cells are characteristically small (less than three times the diameter of a resting lymphocyte), with round, oval, or spindle shapes, scant cytoplasm, and ill-defined borders. The nuclear features are paramount for diagnosis: hyperchromatic nuclei with finely dispersed, granular chromatin, often described as a “salt and pepper” pattern, and absent or inconspicuous nucleoli. A high mitotic rate, extensive necrosis, and prominent nuclear molding (where nuclei deform against one another in tightly packed clusters) are constant findings. The Azzopardi effect, a pathognomonic feature characterized by the basophilic encrustation of blood vessel walls with DNA debris from necrotic tumor cells, is also frequently observed [2,3,6,7,8].

Immunohistochemical (IHC) staining is critical to confirm the diagnosis and distinguish SCLC from other malignancies like lymphoma or non-small-cell lung cancer (NSCLC). SCLC cells are typically positive for neuroendocrine markers, including synaptophysin, chromogranin A, and neural cell adhesion molecule (NCAM/CD56). Thyroid transcription factor-1 (TTF-1) is expressed in approximately 90% of cases and is particularly useful for confirming a pulmonary origin when metastasis is the presenting feature. A high Ki-67 proliferation index, often exceeding 80%, is a hallmark of this rapidly dividing cancer and helps differentiate it from lower-grade neuroendocrine tumors like carcinoids (Table 1) [3,5,6,7,8].

### 2.2. Genomic Landscape and Molecular Drivers

The genomic landscape of SCLC is dominated by the near-universal, biallelic inactivation of two critical tumor suppressor genes: *TP53* and *RB1* (retinoblastoma) (Table 1) [9]. These genetic lesions are present in virtually all SCLCs and are considered the central, foundational events in their carcinogenesis. The loss of these master regulators of the G1/S cell cycle checkpoint removes the normal brakes on cellular proliferation, directly accounting for the extremely high mitotic index and aggressive clinical course of the disease [9,10,11].

This core biological defect also explains the paradoxical clinical behavior of SCLC: its profound initial sensitivity to cytotoxic therapy. Functional p53 and Rb proteins are essential for sensing DNA damage and initiating either cell cycle arrest to allow for repair or apoptosis (programmed cell death) if the damage is irreparable. In SCLC cells lacking these gatekeepers, exposure to DNA-damaging agents like platinum chemotherapy or ionizing radiation does not trigger these protective mechanisms. Instead, the cells are forced to proceed through the cell cycle with catastrophic levels of DNA damage, culminating in mitotic catastrophe and cell death [10,11]. Therefore, the very genetic alterations that make SCLC so aggressive also create a profound vulnerability, explaining the dramatic tumor responses often seen with initial treatment. This also provides a rationale for why resistance develops so quickly, as any clone that survives this initial onslaught has likely evolved mechanisms to bypass these fundamental cell death pathways [12].

Beyond *TP53* and *RB1* loss, other recurrent genomic alterations include deletions on the short arm of chromosome 3 (3p) and amplification of *MYC* family oncogenes (*MYC*, *MYCL*, *MYCN*), which are found in about 30% of tumors and contribute to their proliferative drive [3].

### 2.3. Molecular Subtyping

Recent research has moved beyond a monolithic view of SCLC, identifying distinct molecular subtypes based on the differential expression of key neuroendocrine transcription factors (Table 1). This classification scheme, which has significant therapeutic implications, includes four primary subtypes [13,14].

#### 2.3.1. SCLC-A (ASCL1)

This is the most common or “classic” subtype, characterized by high expression of the transcription factor *ASCL1*. These tumors often exhibit a pure SCLC histology and high expression of neuroendocrine markers [13,14].

#### 2.3.2. SCLC-N (NEUROD1)

Characterized by high expression of *NEUROD1*, this subtype is enriched in combined SCLC/NSCLC histologies and is associated with a higher propensity for metastasis [13,14].

#### 2.3.3. SCLC-P (POU2F3)

This is a less common, non-neuroendocrine variant defined by the expression of *POU2F3* and low or absent expression of neuroendocrine markers [13,14].

#### 2.3.4. SCLC-I (Inflamed)

This subtype is defined by an “inflamed” gene signature, with increased T-cell infiltration and expression of immune checkpoint molecules like PD-L1. It is characterized by low or no expression of the canonical neuroendocrine transcription factors and is hypothesized to be the most responsive to immunotherapy [13,14,15].

## 3. Staging Paradigms

The staging of SCLC is unique among solid tumors, historically relying on a pragmatic, treatment-oriented system that dictates the primary therapeutic approach [16].

### 3.1. The Veterans Administration Lung Study Group (VALSG) Two-Stage System

For decades, the VALSG system has been the clinical standard for staging SCLC, dividing the disease into two categories based on whether it can be treated with curative-intent radiation [17].

#### 3.1.1. Limited-Stage SCLC (LS-SCLC)

This is defined as cancer that is confined to one side of the chest (a single hemithorax) and can be safely encompassed within a single, tolerable radiation therapy port. This includes the primary tumor and may involve lymph nodes in the center of the chest (mediastinum) or above the collarbone (supraclavicular) on the same side as the tumor. Approximately one-third of patients are diagnosed with LS-SCLC [17,18].

#### 3.1.2. Extensive-Stage SCLC (ES-SCLC)

This is defined as disease that has spread too widely to be included in a single radiation field. This includes spread to the other lung, to lymph nodes on the contralateral side of the chest, or to distant organs (e.g., brain, liver, bones, adrenal glands) or the presence of malignant pleural or pericardial effusions [18,19].

## 4. Staging Workup

Accurate staging is critical to ensure that patients receive the appropriate therapy. The standard workup is comprehensive and should be expedited. It includes a history and physical exam, laboratory studies (complete blood count, electrolytes, liver and kidney function tests), contrast-enhanced computed tomography (CT) of the chest and abdomen, and brain imaging. Brain magnetic resonance imaging (MRI) is strongly preferred over CT due to its superior sensitivity and specificity for detecting small brain metastases, which are common in SCLC. A positron emission tomography (PET)/CT scan from the skull base to mid-thigh is crucial for staging and treatment planning in LS-SCLC, as it can identify occult distant metastases that would upstage the patient to ES-SCLC and alter the treatment plan from curative to palliative intent [12,13,14,15,16].

## 5. Management of Limited-Stage SCLC (LS-SCLC): A Curative-Intent Paradigm

**Figure 1:**
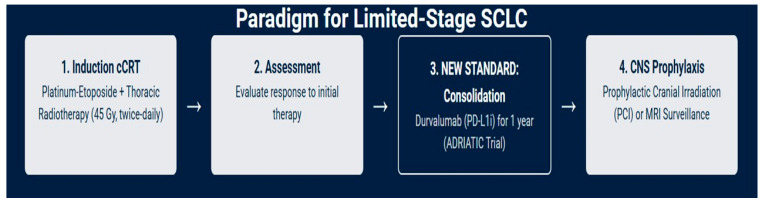
Current management of LS-SCLC: a curative-intent treatment paradigm.

For approximately one-third of patients who present with LS-SCLC, the treatment goal is curative. The therapeutic strategy is aggressive, built upon a foundation of combined-modality therapy (Figure 1) [18,19].

### 5.1. The Cornerstone of Concurrent Chemoradiotherapy (cCRT): Evidence and Regimens

The standard of care for medically fit patients with LS-SCLC is in some cases concurrent chemoradiotherapy (cCRT), a strategy that has remained largely unchanged for decades due to its proven efficacy; in select cases, there may be a possible role for surgery, depending on factors such as performance status, age, and multidisciplinary team evaluation. The rationale for combining modalities is to leverage the systemic activity of chemotherapy to eradicate micrometastatic disease while using radiation to achieve durable local control of the primary tumor and regional lymph nodes [20,21].

Multiple meta-analyses have definitively established the superiority of this concurrent approach. Compared to chemotherapy administered alone, the addition of thoracic radiotherapy (TRT) has been shown to double local tumor control rates (from ~25% to ~50%) and provide a small but significant improvement in 3- and 5-year overall survival rates [22,23,24,25].

#### 5.1.1. Chemotherapy Backbone

The most widely used and validated chemotherapy backbone for cCRT is a two-drug combination of a platinum agent and etoposide (EP) for four to six cycles [22,24].

#### 5.1.2. Cisplatin vs. Carboplatin

Cisplatin plus etoposide is the most established regimen with the longest track record of evidence [26]. However, in clinical practice, carboplatin is frequently substituted for cisplatin to reduce the risk of significant non-hematologic toxicities, including nephrotoxicity, ototoxicity, peripheral neuropathy, and severe nausea and vomiting. This improved tolerability must be balanced against a greater risk of myelosuppression with carboplatin. A meta-analysis of four randomized trials including 663 SCLC patients (32% with LS-SCLC) found no significant difference in response rate, progression-free survival (PFS), or overall survival (OS) between cisplatin- and carboplatin-containing regimens, supporting their interchangeability based on patient comorbidities and toxicity profiles [27].

#### 5.1.3. Alternative Regimens

Other combinations, such as irinotecan plus cisplatin (IP), have been investigated, particularly in Asian populations where it has shown efficacy [28]. However, a large-scale, nationwide retrospective cohort study from Korea directly comparing EP-cCRT with IP-cCRT found that the EP-based regimen resulted in significantly more favorable OS (median 22.2 months vs. 16.4 months) and time to first subsequent therapy (11.2 months vs. 9.6 months), reinforcing EP as the preferred global standard [29].

#### 5.1.4. Timing of cCRT

The timing of radiation relative to chemotherapy is critical. Evidence strongly supports initiating TRT early and concurrently with chemotherapy, ideally with the first or second cycle, as this approach yields superior survival outcomes compared to a sequential strategy where radiation is delivered after the completion of all chemotherapy cycles [30,31].

### 5.2. Optimizing Thoracic Radiotherapy: Dose, Fractionation, and Technology

The delivery of TRT in LS-SCLC has been the subject of extensive investigation, with a focus on optimizing the dose and fractionation schedule to maximize tumor cell kill while minimizing damage to surrounding normal tissues like those in the lungs, esophagus, and heart [32].

#### 5.2.1. Dose and Fractionation

The central debate in TRT for LS-SCLC has been between hyperfractionated (twice-daily) and conventional (once-daily) schedules [32].

#### 5.2.2. Standard Regimen

A landmark intergroup trial established that a hyperfractionated, accelerated regimen of 45 Gy delivered in 1.5 Gy twice-daily fractions over three weeks is superior to a once-daily regimen of 45 Gy in 1.8 Gy fractions over five weeks. This twice-daily approach is considered the standard of care by NCCN and other international guidelines [32,33,34]. When this schedule is used, a minimum interval of six hours between fractions is essential to allow for the repair of sublethal damage in normal tissues [30].

#### 5.2.3. Alternative Regimen

For patients or centers where twice-daily treatment is not feasible, a once-daily regimen is an acceptable alternative. However, to achieve a biologically equivalent dose, higher total doses of 60–70 Gy are recommended. It is important to note that two large phase III trials, CONVERT and CALGB 30610, failed to demonstrate a survival advantage for high-dose (60–70 Gy) once-daily TRT compared to the 45 Gy twice-daily standard, reinforcing the latter as the preferred regimen when possible [32,33,34,35].

#### 5.2.4. Prophylactic Cranial Irradiation (PCI)

SCLC exhibits a profound tropism for the central nervous system (CNS), with over 50% of patients developing brain metastases (BMs) within two years of diagnosis if no preventive measures are taken [36,37]. Prophylactic cranial irradiation (PCI) is a strategy designed to sterilize occult micrometastases within the brain before they become clinically apparent [37,38,39,40]

##### Efficacy in LS-SCLC

The role of PCI in LS-SCLC was established by a pivotal meta-analysis of individual patient data from seven randomized trials [41]. This analysis demonstrated that for patients who achieved a complete or partial response to initial cCRT, PCI significantly reduced the cumulative 3-year incidence of BM from 58.6% in the observation group to 33.3% in the PCI group. Crucially, this reduction in central nervous system (CNS) relapse translated into a significant overall survival benefit, improving the 3-year OS rate by an absolute 5.4% (from 15.3% to 20.7%). This Level I evidence cemented PCI as a standard of care for responding LS-SCLC patients for over two decades [41,42,43,44,45].

##### PCI Dose

**Figure 2:**
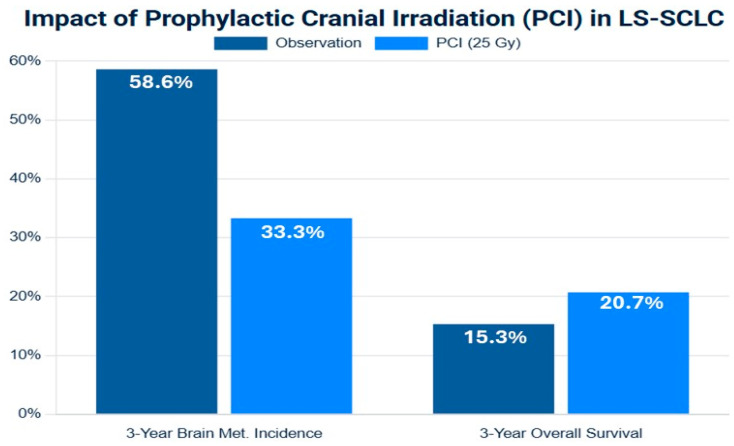
Forest plot of meta-analysis of prophylactic cranial irradiation (PCI) versus observation in limited-stage small-cell lung cancer (LS-SCLC) after response to initial therapy. The plot illustrates the hazard ratios (HRs) and 95% confidence intervals (CIs), in the left side brain metastasis-free survival (BMFS), and in the write side the overall survival (OS). The summary estimate (diamond) demonstrates a consistent and significant reduction in brain metastasis (HR < 1) and a more modest but statistically significant improvement in overall survival. Data are synthesized from representative studies included in meta-analyses [41,48].

The standard, evidence-based dose for PCI is 25 Gy delivered in 10 daily fractions over two weeks. This was established by a large Phase III trial led by Le Péchoux et al., which compared this dose to a higher dose of 36 Gy. The trial found no difference in the 2-year incidence of BM between the arms (29% vs. 23%) but observed a significant increase in treatment-related mortality in the higher-dose arm, making 25 Gy the safer and equally effective standard (Figure 2) [42,43,44,45,46,47].

The following (Figure 2) synthesizes data from meta-analyses to visually represent the evidence for PCI in LS-SCLC.

### 5.3. The MRI-Era Controversy and Neurotoxicity

In recent years, the established role of PCI has been challenged by two key factors: the routine use of high-sensitivity brain MRI for surveillance and a greater appreciation for the long-term neurocognitive toxicity of cranial irradiation [44].

#### 5.3.1. Impact of MRI

The original trials establishing PCI’s benefit were conducted in an era where brain imaging was not routinely performed prior to randomization. More recent meta-analyses that include studies where patients were screened with MRI to rule out existing metastases prior to PCI have shown a diminished or even absent overall survival benefit, although the significant reduction in the incidence of brain metastases persists. This suggests that for some patients, a strategy of active MRI surveillance with subsequent stereotactic radiosurgery (SRS) for any detected oligo-metastases may yield similar survival outcomes without the upfront toxicity of irradiating the entire brain [44].

#### 5.3.2. Neurotoxicity

PCI is associated with a significant risk of chronic neurocognitive decline, particularly affecting memory, learning, and executive function, which can substantially impair a patient’s quality of life. This risk is a primary driver for seeking alternatives to universal PCI [45].

#### 5.3.3. Hippocampal Avoidance (HA-PCI):

To mitigate this toxicity, techniques have been developed to spare the hippocampi, which are critical for memory function but are a rare site of SCLC metastasis. Phase III trials have yielded mixed results: the PREMER trial showed that HA-PCI reduced the decline in delayed free recall compared to standard PCI without increasing the rate of brain failure [46]. However, a similar trial did not find a significant difference in cognitive decline. HA-PCI remains an area of active investigation and is considered an option by NCCN guidelines, particularly for patients concerned about cognitive side effects [46,48].

### 5.4. Current Treatment Options for Limited-Stage SCLC

The Phase III ADRIATIC trial evaluated maintenance treatment with durvalumab—with or without tremelimumab—versus placebo in patients with limited-stage small-cell lung cancer (LS-SCLC) who had not progressed after cCRT. In the first planned interim analysis (data cutoff 15 January 2024), durvalumab monotherapy significantly improved both overall survival (median, 55.9 months vs. 33.4 months; HR, 0.73; *p* = 0.01) and progression-free survival (median, 16.6 months vs. 9.2 months; HR, 0.76; *p* = 0.02) compared to placebo. Safety data showed grade 3–4 adverse events in about 24% of patients, with similar rates of pneumonitis between arms [49].

### 5.5. Current Ongoing Clinical Trials for Limited-Stage SCLC (Table 2)

The Current Ongoing Clinical Trials for Limited-Stage SCLC are mentioned in Table 2.

## 6. Extensive-Stage SCLC

**Figure 3:**
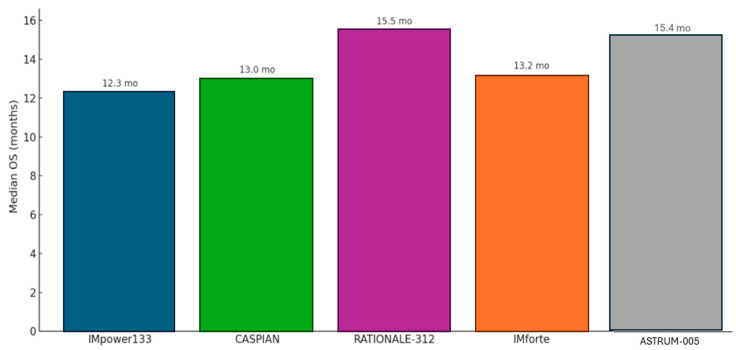
Median overall survival for current immune checkpoint inhibitors in first-line therapy for ES-SCLC.

For the two-thirds of patients presenting with ES-SCLC, the treatment intent is palliative, aimed at controlling symptoms, improving quality of life, and prolonging survival. For decades, the undisputed standard of care was four to six cycles of platinum–etoposide (EP) chemotherapy, a regimen that reliably produced high initial response rates but offered a grimly predictable median OS of only about 10 months (mo). The introduction of immune checkpoint inhibitors has, for the first time, broken through this long-standing therapeutic ceiling (Figure 3) [50,51,52].

### 6.1. Management of First Line of Therapy for Extensive-Stage SCLC

#### 6.1.1. IMpower133 Trial (Atezolizumab)

The IMpower133 trial was the first landmark global, randomized, double-blind, placebo-controlled Phase III study that established the role of chemoimmunotherapy in the treatment of ES-SCLC. This pivotal trial evaluated the efficacy and safety of combining the PD-L1 inhibitor atezolizumab with standard chemotherapy—carboplatin and etoposide (CP/ET)—compared to placebo plus CP/ET over four induction cycles, followed by maintenance atezolizumab or placebo. The trial met both of its co-primary endpoints, showing a statistically significant improvement in overall survival (OS) and progression-free survival (PFS). Median OS was 12.3 months in the atezolizumab arm versus 10.3 months in the control arm (HR, 0.70; 95% CI, 0.54–0.91; *p* = 0.007), while median PFS was 5.2 months versus 4.3 months, respectively (HR, 0.77; 95% CI, 0.62–0.96; *p* = 0.02). Updated analyses with a median follow-up of 22.9 months confirmed a durable OS benefit (HR, 0.76) and an improved 18-month survival rate (34.0% vs. 21.0%), reflecting a meaningful “tail of the curve” effect. The addition of atezolizumab was associated with a manageable safety profile, with immune-related adverse events occurring at expected rates consistent with prior data. Crucially, patient-reported outcomes demonstrated no deterioration in health-related quality of life, underscoring that the survival advantage was achieved without additional patient burden. This trial firmly established chemoimmunotherapy with atezolizumab as a new standard of care in ES-SCLC [52,53,54].

#### 6.1.2. CASPIAN Trial (Durvalumab)

The CASPIAN trial, a global, randomized, open-label Phase III study, provided strong confirmatory evidence supporting the chemoimmunotherapy approach in ES-SCLC. This three-arm trial compared standard EP alone to EP combined with the PD-L1 inhibitor durvalumab, and to a triplet regimen of durvalumab, the CTLA-4 inhibitor tremelimumab, and EP. Unlike IMpower133, CASPIAN offered greater treatment flexibility, allowing investigator choice of platinum agent (cisplatin or carboplatin) and up to six chemotherapy cycles. The durvalumab plus EP arm met its primary endpoint, significantly improving overall survival compared to EP alone (median OS, 13.0 vs. 10.3 months; HR, 0.73; 95% CI, 0.59–0.91; *p* = 0.0047), along with a higher objective response rate (68% vs. 58%). Long-term follow-up analyses at 25.1 and 39.4 months confirmed a sustained and even deepening survival benefit (HRs of 0.75 and 0.71, respectively), with the 3-year OS rate nearly tripling in the durvalumab arm (17.6%) compared to that with chemotherapy alone (5.8%), highlighting a durable response in a subset of patients. However, the addition of tremelimumab did not confer a statistically significant OS advantage over chemotherapy alone (median OS, 10.4 vs. 10.5 months; HR, 0.82; *p* = 0.045), and as such, the triplet regimen is not considered a standard of care. These results further solidify durvalumab plus EP as an effective frontline option in ES-SCLC treatment [55].

#### 6.1.3. RATIONALE-312 Trial (Tislelizumab)

The RATIONALE-312 trial was a pivotal, multicenter, double-blind, placebo-controlled Phase III study designed to evaluate the efficacy and safety of tislelizumab, an anti–PD-1 monoclonal antibody, in combination with chemotherapy as a first-line treatment for patients with previously untreated ES-SCLC. Conducted primarily in China, the trial enrolled 457 patients between July 2019 and April 2021, who were randomized 1:1 to receive either tislelizumab or placebo, both in combination with etoposide and a platinum agent (cisplatin or carboplatin) for four cycles, followed by maintenance therapy with either tislelizumab or placebo. The primary endpoint was OS, with PFS, the objective response rate (ORR), and safety as key secondary endpoints.

The trial demonstrated significant clinical benefit from the addition of tislelizumab. Median OS was 15.5 months in the tislelizumab arm versus 13.5 months in the placebo arm, corresponding to a 25% reduction in the risk of death (HR = 0.75; 95% CI: 0.61–0.93; *p* = 0.0040). Tislelizumab also significantly prolonged PFS, with a median of 4.7 months compared to 4.3 months in the placebo group (HR = 0.64; 95% CI: 0.52–0.78; *p* < 0.0001). Estimated PFS rates at 6 and 12 months were notably higher in the tislelizumab arm (35% and 21%, respectively) versus in the placebo (18% and 5%). Additionally, the confirmed ORR was higher in the tislelizumab group at 68% versus 62%, with a longer median duration of response (4.3 vs. 3.7 months). With extended follow-up (minimum 23.9 months), the trial also demonstrated a durable long-term benefit, evidenced by improved 2-year survival probability and a high event-to-patient ratio (78%) for OS analysis [56].

#### 6.1.4. ASTRUM-005 Trial (Serplulimab)

The ASTRUM-005 trial was an international, multicenter, double-blind, randomized, placebo-controlled Phase 3 study that has significantly advanced the first-line treatment ES-SCLC. The primary objective was to evaluate the efficacy and safety of adding the anti-PD-1 antibody serplulimab to standard chemotherapy (carboplatin and etoposide) compared to chemotherapy plus a placebo. The trial enrolled 585 previously untreated patients with ES-SCLC from 114 sites across multiple countries, including a notable proportion of non-Asian participants, enhancing its global relevance.

The study successfully met its primary endpoint of OS, demonstrating a statistically significant and clinically meaningful benefit for the serplulimab combination. Patients receiving serplulimab achieved a median OS of 15.4 months compared to 10.9 months in the placebo group, representing a substantial reduction in the risk of death. This benefit was supported by an improvement in PFS, which was 5.7 months in the serplulimab arm versus 4.3 months in the placebo arm. The safety profile of the serplulimab combination was manageable and consistent with the known side effects of immunotherapy and chemotherapy, with no unexpected safety concerns arising.

The significance of ASTRUM-005 lies in it being the first global Phase 3 study to validate a PD-1 inhibitor in this setting—previous successful trials had used PD-L1 inhibitors—leading to regulatory approvals and establishing a new standard of care for ES-SCLC [57].

#### 6.1.5. IMforte Trial (New Strategy for Induction Phase: Atezolizumab Monotherapy or a Combination of Atezolizumab and Lurbinectedin)

The IMforte trial was a landmark Phase III, open-label, randomized, multicenter study; unlike prior studies such as IMpower133, CASPIAN, and RATIONALE-312, which focused on first-line combination therapies, IMforte uniquely investigated the role of maintenance therapy following standard induction treatment [51,52,53,54,55,56]. A total of 660 patients with previously untreated ES-SCLC—excluding those with brain or spinal cord metastases—received four 21-day cycles of atezolizumab, carboplatin, and etoposide. Of these, 483 patients who did not experience disease progression were randomized 1:1 into the maintenance phase to receive either atezolizumab monotherapy or a combination of atezolizumab and lurbinectedin. Treatment continued until disease progression, unacceptable toxicity, or patient withdrawal. The study’s primary endpoints were PFS and OS, assessed from the point of randomization into maintenance therapy.

The IMforte trial demonstrated statistically significant and clinically meaningful improvements in both OS and PFS with the combination therapy. Median OS was 13.2 months in the combination arm compared to 10.6 months in the atezolizumab monotherapy arm, reflecting a 27% reduction in the risk of death (HR = 0.73; 95% CI: 0.57–0.95; *p* = 0.0174). PFS was also significantly prolonged—5.4 months vs. 2.1 months—representing a 46% reduction in the risk of progression or death (HR = 0.54; 95% CI: 0.43–0.67; *p* < 0.0001). Six- and twelve-month PFS rates were 41.2% and 20.5% in the combination arm, respectively, compared to 18.7% and 12.0% with atezolizumab alone.

The combination of lurbinectedin and atezolizumab was generally well-tolerated, with no new or unexpected safety signals. However, treatment-related adverse events were more frequent in the combination arm (83.5%) versus those under monotherapy (40%), with Grade 3/4 events—primarily anemia, neutropenia, and thrombocytopenia—occurring in 25.6% and 5.8% of patients, respectively. Treatment discontinuation due to toxicity was also slightly higher in the combination group (6.2% vs. 3.3%). Despite these increased toxicities, the benefit–risk balance remains favorable. In conclusion, the IMforte trial provides compelling evidence that maintenance therapy with lurbinectedin plus atezolizumab significantly extends survival and disease control in patients with ES-SCLC post-induction therapy, representing a major advance in addressing the unmet need in this aggressive disease (Table 3) [58].

Below is a detailed comparison table summarizing key features of the IMpower133, CASPIAN, RATIONALE-312, ASTRUM-005, and IMforte studies (Table 3) [51,52,53,54,55,56].

**Table 3:** Showing the summarizing key features of the IMpower133, CASPIAN, RATIONALE-312, ASTRUM-005, and IMforte studies.

Feature	IMpower133 [53,54]	CASPIAN [55]	RATIONALE-312 [56]	ASTRUM-005 [57]	IMforte [58]
Study Design	First-line induction	First-line induction	First-line induction	First-line induction	First-line maintenance (after chemoimmunotherapy induction)
Study Drug(s)	Atezolizumab + carboplatin + etoposide	Durvalumab + platinum + etoposide (± tremelimumab)	Tislelizumab + platinum (carboplatin or cisplatin) + etoposide	Serplulimab + carboplatin + etoposide	Lurbinectedin + atezolizumab vs. atezolizumab alone (maintenance after platinum–etoposide–atezo)
Primary Endpoint	OS and PFS (co-primary)	OS	OS	OS	OS and IRF-assessed PFS (co-primary, from randomization into maintenance)
OS (Median)	12.3 months (vs. 10.3 months for chemo alone)	13.0 months (vs. 10.3 months for chemo alone) (durvalumab + EP arm)	15.5 months (vs. 13.5 months for chemo alone)	15.4 months (vs. 10.9 months for chemo alone)	13.2 months (lurbinectedin + atezo) vs. 10.6 months (atezo alone)
PFS (Median)	5.2 months (vs. 4.3 months for chemo alone)	5.1 months (durvalumab + EP arm; vs. 5.4 months chemo alone)	6.9 months (vs. 4.2 months for chemo alone)	5.7 months (vs. 4.3 months for chemo alone)	5.4 months (lurbinectedin + atezo) vs. 2.1 months (atezo alone)
Patients Enrolled	403	805 (randomized to 3 arms)	457	585	483 (maintenance phase; 660 patients initially enrolled in induction phase)
Most Common AEs (Grade 3/4)	Neutropenia, anemia, thrombocytopenia, febrile neutropenia, fatigue	Neutropenia, anemia, thrombocytopenia, leukopenia	Neutropenia, ↓ WBC count, anemia, thrombocytopenia, alopecia, nausea	Neutropenia, ↓ WBC count, anemia, thrombocytopenia	Higher AE incidence in combo arm (83.5% vs. 40%); Grade 3/4 AEs: 25.6% (combo) vs. 5.8% (atezo alone). Specific AEs not detailed.

Abbreviations: atezolizumab, atezo; chemotherapy, chemo; OS, overall survival; adverse events, AEs; progression-free survival, PFS; platinum agent and etoposide, EP; white blood cells, WBCs; ↓, low.

### 6.2. Therapeutic Approaches Following Disease Progression in Extensive-Stage SCLC

Despite the improvements in first-line therapy, disease progression is an unfortunate inevitability for nearly all patients with ES-SCLC. The choice of subsequent therapy is guided primarily by the duration of the response to first-line platinum-based treatment, known as the chemotherapy-free interval (TFI).

#### 6.2.1. Platinum-Sensitive Relapse (TFI > 6 Months)

For patients who experience disease progression more than 6 months after completing initial platinum-based therapy, re-challenging with the same platinum–etoposide regimen is a standard and effective option [59].

#### 6.2.2. Platinum-Resistant/-Refractory Relapse (TFI < 6 Months)

This setting represents a major clinical challenge with limited effective options [60].

#### 6.2.3. Lurbinectedin

The FDA approval of lurbinectedin, a selective inhibitor of oncogenic transcription as well as DNA damaging agent, provided a significant new option for second-line therapy. In a pivotal single-arm trial, lurbinectedin demonstrated an ORR of 35.2% and a median OS of 9.3 months in patients who had progressed after one prior platinum-containing line. A post hoc analysis suggested it has superior efficacy and a more favorable toxicity profile (particularly less hematologic toxicity) compared to topotecan [61].

#### 6.2.4. Topotecan

For many years, topotecan was the only approved second-line agent. It remains an option but is often limited by significant myelosuppression and modest efficacy [60,62].

#### 6.2.5. Tarlatamab

**Figure 4:**
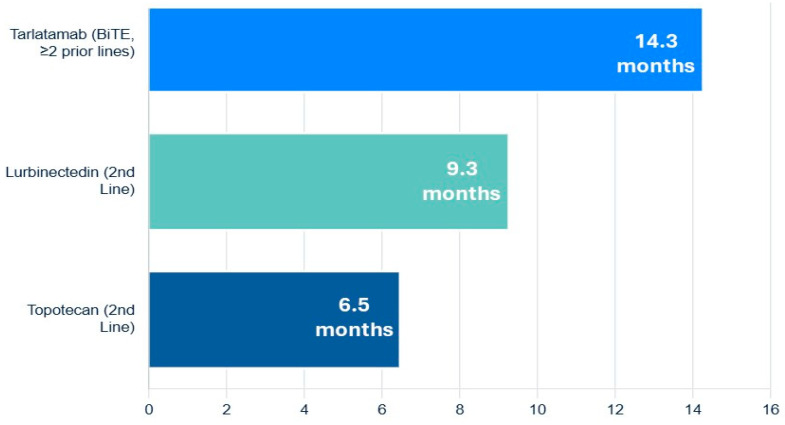
Navigating the overall survival of relapsed or refractory extensive-stage small-cell lung cancer.

A groundbreaking advance in the relapsed/refractory setting is the recent accelerated FDA approval of tarlatamab, a Bi-specific T-cell Engager (BiTE) antibody that targets both delta-like ligand 3 (DLL3) on SCLC cells and CD3 on T-cells. By physically linking T-cells to tumor cells, it forces a potent, targeted immune attack. In the DeLLphi-301 trial of heavily pre-treated patients (median of two prior lines of therapy), tarlatamab produced an impressive ORR of 40% and a median OS of 14.3 months, establishing a new, highly active therapeutic class for this disease (Figure 4) [63,64].

#### 6.2.6. Clinical Trials

Given the poor outcomes associated with relapsed SCLC, enrolment in clinical trials investigating novel agents and combinations is strongly encouraged at every stage of the disease.

### 6.3. The Role of Consolidative Thoracic Radiotherapy in the Immunotherapy Era

The role of TRT in ES-SCLC is less defined than that in LS-SCLC. In the pre-immunotherapy era, the Phase III CREST trial investigated consolidative TRT to the chest in patients who had responded to initial chemotherapy. While the trial did not meet its primary endpoint, a post hoc analysis revealed an improvement in 2-year OS (from 3% to 13%), particularly in the subgroup of patients with residual disease confined to the thorax after chemotherapy [65].

The pivotal chemoimmunotherapy trials IMpower133 and CASPIAN did not permit the use of consolidative TRT, leaving its role in the modern era as a critical unanswered question. There is a strong rationale that local consolidation with radiation could synergize with systemic immunotherapy by eliminating resistant clones and stimulating a further anti-tumor immune response. This question is being directly addressed in the ongoing Phase II/III RAPTOR trial (NRG-LU007), which is randomizing ES-SCLC patients who respond to induction chemoimmunotherapy to receive either consolidative TRT plus maintenance immunotherapy or maintenance immunotherapy alone. The results of this trial are highly anticipated and have the potential to establish a new standard of care for a subset of ES-SCLC patients [66].

### 6.4. Management of Brain Metastases

#### 6.4.1. The Evolving Role of PCI and MRI Surveillance in ES-SCLC

The utility of PCI in ES-SCLC is more contentious than that in LS-SCLC. An early European trial demonstrated that in patients who responded to initial chemotherapy, PCI reduced the rate of symptomatic BM and improved 1-year OS but did not improve the median OS. A subsequent Japanese trial, which was more stringent in its design by mandating a baseline brain MRI to exclude pre-existing metastases, found that PCI offered no OS benefit at all [67].

Given this conflicting evidence and the known risk of neurotoxicity, current NCCN and ESMO guidelines suggest that PCI can be *considered* for ES-SCLC patients who have a good response to first-line systemic therapy but that active surveillance with serial brain MRIs is an equally acceptable alternative. The decision should be individualized through a process of shared decision-making, weighing the potential reduction in CNS relapse against the risks of cognitive decline [33].

The introduction of chemoimmunotherapy has added another layer to this discussion. Both the IMpower133 and CASPIAN trials allowed the inclusion of patients with treated, stable brain metastases, and subgroup analyses have consistently shown that the survival benefit of adding a PD-L1 inhibitor is maintained in this population. Furthermore, an exploratory analysis of the IMpower133 trial suggested that the addition of atezolizumab may delay the time to intracranial progression, hinting at potential CNS activity of the chemoimmunotherapy combination [53,55].

#### 6.4.2. Indication for Surgical Intervention

The decision to pursue surgical intervention for brain metastases is never made in isolation but rather arises from a comprehensive, multidisciplinary tumor board discussion involving a neurosurgeon, medical oncologist, and radiation oncologist. This collaborative process carefully weighs the mechanical benefits of surgical decompression against a patient’s overall prognosis, functional status, and the optimal timing of systemic and radiation therapies. Neurosurgery, in this context, often functions as a critical bridge—addressing an immediate, life-threatening neurological crisis to stabilize the patient and enable timely delivery of definitive oncologic treatments [64]. The primary role of surgery is to manage acute neurological compromise. For instance, a patient presenting with a large, solitary brain metastasis causing significant mass effect—manifesting as midline shift on imaging and symptoms such as hemiparesis or aphasia—requires urgent neurosurgical evaluation. In such cases, systemic therapies and radiation are insufficiently rapid to relieve intracranial pressure. Surgical debulking offers both life-saving and function-preserving benefits, rapidly alleviating mass effect and improving neurologic function, thereby facilitating patients’ transition to further cancer-directed therapies [68,69].

##### Management of Hemorrhagic Metastases

SCLC brain metastases have a tendency to bleed. An acute intra-tumoral hemorrhage can lead to a rapidly expanding hematoma, increasing intracranial pressure and risking brain herniation. Emergency surgical evacuation of the hematoma is often required in these cases [68,69].

##### Diagnostic Uncertainty

In the rare clinical scenario where a patient presents with a solitary brain lesion as the only evidence of disease and the primary lung tumor is not apparent, a neurosurgical procedure—either a stereotactic biopsy or a complete resection—is necessary to obtain a tissue diagnosis. This pathological confirmation is essential to guide all subsequent systemic and radiation therapy [70,71,72].

### 6.5. Current Ongoing Clinical Trials for Extensive-Stage SCLC (Table 4)

## 7. Discussion

SCLC is accounts for approximately 10–15% of all lung cancers and is strongly associated with smoking, presenting most often at an advanced stage with limited therapeutic options and a generally poor prognosis. SCLC is an aggressive, high-grade neuroendocrine tumor characterized by rapid growth, early metastasis, and a strong initial response to chemotherapy and radiotherapy, yet it often relapses quickly with poor long-term survival [1,2,3].

The management of SCLC has entered a new and dynamic era. For patients with limited-stage disease, concurrent chemoradiotherapy remains the cornerstone of curative-intent treatment, with the optimal radiation schedule being 45 Gy in twice-daily fractions. The landmark results of the ADRIATIC trial are set to establish consolidative durvalumab as a new standard of care following cCRT, finally breaking a decades-long therapeutic stalemate. The role of prophylactic cranial irradiation, while still beneficial in reducing brain metastases, is being increasingly challenged by the availability of high-sensitivity MRI surveillance and a greater focus on mitigating long-term neurocognitive toxicity, necessitating individualized, shared decision-making [23,26,28,40,49].

The current treatment paradigm for ES-SCLC, a notoriously aggressive malignancy with historically grim prognoses, has been profoundly reshaped by the integration of immunotherapy. For decades, platinum–etoposide chemotherapy remained the sole standard, but a series of pivotal clinical trials, including IMpower133, CASPIAN, RATIONALE-312, ASTRUM-005, and, more recently, IMforte, have firmly established chemo-immunotherapy as the new first-line standard and introduced the promising concept of maintenance therapy [53,54,55,56,57,58].

IMpower133, a foundational study, demonstrated the initial and critical benefit of adding atezolizumab, a PD-L1 inhibitor, to standard platinum–etoposide chemotherapy in the first-line setting for ES-SCLC. This trial marked a paradigm shift, showing a modest yet clinically meaningful improvement in OS and PFS. Its findings were significant because they offered the first real breakthrough in decades for this challenging disease, showcasing a manageable safety profile and, importantly, a longer “tail” to the survival curve, indicating a subset of patients achieving more durable responses. Subsequent real-world data have largely corroborated IMpower133′s efficacy and safety, affirming its broader applicability even in patients with characteristics that might have excluded them from the original trial [53,54].

Building on this success, the CASPIAN trial further solidified the role of immunotherapy by demonstrating the efficacy of durvalumab, another anti-PD-L1 antibody, in combination with platinum–etoposide chemotherapy. CASPIAN’s design was notable for also exploring a triplet regimen that included tremelimumab, an anti-CTLA-4 antibody. While durvalumab plus chemotherapy proved beneficial, the addition of tremelimumab did not confer a statistically significant incremental OS benefit, an important finding that guides the selection of dual checkpoint inhibition strategies in this context. Long-term follow-up from CASPIAN has reinforced the sustained survival benefit of durvalumab plus chemotherapy, with three times more patients estimated to be alive at 3 years compared to those under chemotherapy alone. Exploratory analyses from CASPIAN also contributed to the ongoing discussion about potential biomarkers, although no definitive predictive markers have emerged to date [55].

Expanding the therapeutic landscape, RATIONALE-312 introduced tislelizumab, an anti-PD-1 antibody, as another effective first-line option when combined with platinum–etoposide chemotherapy for ES-SCLC. Beyond demonstrating comparable efficacy to the anti-PD-L1 agents, this trial notably presented a cost-effectiveness analysis from a Chinese healthcare system perspective, highlighting the potential for broader accessibility and integration into diverse clinical settings. The RATIONALE-312 trial, with a median follow-up of 14.2 months, showed a median OS of 15.5 months with tislelizumab plus chemotherapy, further substantiating the benefit of PD-1 inhibition [56].

ASTRUM-005, evaluating serplulimab, another anti-PD-1 antibody, in combination with chemotherapy, reported data on some of the longest median OSs for first-line immunotherapy in ES-SCLC, with a median OS of 15.8 months. Updated analyses with longer follow-up (42.4-month median follow-up) showed a 4-year OS rate of 21.9%, an impressive figure for this aggressive disease. This trial also ventured into novel biomarker exploration, identifying a 15-protein signature and specific gene mutations (RB1 or Notch pathway) as potentially predictive of benefit, while the baseline neutrophil-to-lymphocyte ratio (NLR) and lactate dehydrogenase (LDH) were identified as prognostic factors. These findings underscore the ongoing efforts to personalize ES-SCLC treatment, a critical unmet need given the lack of robust predictive biomarkers currently [57].

Most recently, IMforte has introduced a groundbreaking concept: maintenance therapy. This trial demonstrated that adding lurbinectedin, a DNA-alkylating agent, to atezolizumab as first-line maintenance therapy, following induction with atezolizumab and platinum–etoposide, significantly improved both PFS and OS compared to atezolizumab maintenance alone. With a median follow-up of 15 months, patients receiving the lurbinectedin–atezolizumab maintenance had a 27% lower risk of death (median OS of 13.2 months vs. 10.6 months), a clinically and statistically significant improvement. This marks a significant evolution in ES-SCLC management, suggesting that continuous active treatment beyond the initial induction phase can further extend survival. The manageable safety profile of this novel combination in the maintenance setting makes it a highly promising strategy to address the high relapse rates characteristic of SCLC [58].

In the relapsed/refractory setting, the approval of targeted agents like the transcription inhibitor lurbinectedin and the DLL3-targeting BiTE tarlatamab have provided crucial new options for patients with this recalcitrant disease [61,63,64].

## 8. Conclusions

Despite its aggressive nature and historically poor prognosis, the treatment landscape of SCLC is undergoing a significant transformation. Advances in treatment for both limited-stage and extensive-stage disease—ranging from optimized chemoradiotherapy protocols to the integration of immunotherapy and targeted agents—are beginning to improve outcomes in meaningful ways. The introduction of consolidative durvalumab after chemoradiotherapy in limited-stage disease and the incorporation of PD-L1 inhibitors into first-line therapy for extensive-stage disease mark critical milestones. Furthermore, emerging therapies in the relapsed setting offer new hope for a disease long considered intractable. Continued research and refinement of therapeutic strategies, guided by multidisciplinary care and personalized decision-making, are essential to sustain and build upon this momentum.

## Figures and Tables

**Table 1:** Pathological and molecular characteristics of SCLC. Data compiled from sources.

Feature Category	Specific Marker/Feature	Typical Finding/Prevalence	Clinical/Biological Significance
	Cell Size	Small (<3x lymphocyte diameter)	Diagnostic criterion
	Cytoplasm	Scant	Diagnostic criterion
	Chromatin	Finely granular (“salt and pepper”)	Diagnostic criterion, distinguishes from NSCLC
***Histology***	Nucleoli	Absent or inconspicuous	Key distinguishing feature from NSCLC
	Mitotic Rate	High (>10 per 10 HPF)	Reflects rapid proliferation
	Nuclear Molding	Present	Diagnostic feature of cellular crowding
	Ki-67 Index	High (>80%)	Hallmark of high-grade proliferation
***Immunohistochemistry***	Synaptophysin, Chromogranin, CD56	Positive	Confirms neuroendocrine differentiation
	TTF-1	Positive (~90%)	Confirms pulmonary origin
	p-RB	Negative/absent	Reflects universal *RB1* gene inactivation
	*TP53* Inactivation	>90%	Loss of key tumor suppressor and apoptosis regulator
***Key genetic alterations***	*RB1* Inactivation	>95%	Loss of key cell cycle gatekeeper
	*Chromosome 3p Deletion*	>90%	Loss of tumor suppressor genes (e.g., FHIT)
	*MYC Family Amplification*	~30%	Drives proliferation
	*SCLC-A*	ASCL1-high	Most common subtype, classic neuroendocrine features
***Molecular subtypes (inflamed signature with absence of ASCL-1, NEUROD1, POU2F3)***	*SCLC-N*	NEUROD1-high	Associated with metastasis and combined histology
	*SCLC-P*	POU2F3-high	Non-neuroendocrine variant, distinct biology
	*SCLC-I*	Inflamed signature	Immune-infiltrated, potential for immunotherapy response

Abbreviations: neural cell adhesion molecule, CD56; SCLC, small-cell lung cancer; thyroid transcription factor 1, TTF-1; retinoblastoma protein, p-RB; tumor suppressor protein, TP53; retinoblastoma 1 gene, RB1; myelocytomatosis, myc; non-small-cell lung cancer, NSCLC; Fragile Histidine Triad Diadenosine Triphosphatase, FHIT; ASCL1, achaete-scute homolog 1; Neurogenic Differentiation Factor 1, NEUROD; POU Class 2 Homeobox 3, POU2F3; high-pass filter, HPF; bold and italics: subtype; background color: asier for understanding each subject separately.

**Table 2:** Showing the current ongoing clinical trials for limited-stage SCLC.

Study Title (NCT Number)	Main Therapeutic Strategy	Simplified Patient Participants	Common Primary Endpoints (Highly Likely)	Reference
Phase 3 Study of Toripalimab Alone or in Combination With Tifcemalimab as Consolidation Therapy	Immunotherapy (Toripalimab ± Tifcemalimab) as consolidation after cCRT	Adults with LS-SCLC, ECOG 0–1, achieved CR/PR/SD after platinum-based cCRT	OS, PFS, Safety	[7,48]
General Research Direction	Immunotherapy (concurrent or consolidation) with chemoradiotherapy; PCI vs. surveillance	LS-SCLC patients post-treatment or in combination with standard therapy	OS, PFS, Brain Metastasis Rate, QoL	[25,49]

Abbreviations: concurrent chemoradiotherapy, cCRT; limited-stage small-cell lung cancer, LS-SCLC; overall survival, OS; progression-free survival, PFS; complete response, CR; partial response, PR; stable disease, SD; prophylactic cranial irradiation, PCI; quality of life, QoL.

**Table 4:** Showing the summarizing the Current Ongoing Clinical Trials for Extensive-Stage SCLC.

Study Title (NCT Number)	Main Therapeutic Strategy	Simplified Patient Participants	Common Primary Endpoints	REF
RAPTOR Trial: Testing Radiation with Atezolizumab (NCT04402788)	Addition of Thoracic Radiation to Atezolizumab	ES-SCLC patients on atezolizumab	OS, PFS, Local Control, Safety	[66]
Iadademstat + Atezolizumab/Durvalumab (NCT06287775)	LSD1 Inhibitor (Iadademstat) + Immunotherapy	SCLC patients (mostly ES-SCLC)	ORR, DOR, PFS, OS, Safety	[73]
Ifinatamab Deruxtecan vs. Physician’s Choice (NCT06203210)	Antibody-Drug Conjugate (ADC) vs. Standard Therapy	Relapsed SCLC patients (ES-SCLC or relapsed LS-SCLC)	OS, PFS, ORR, Safety	[74]
IDeate-Lung03: I-DXd + Atezolizumab ± Carboplatin (NCT06362252)	ADC (I-DXd) + Atezolizumab ± Carboplatin (1L Induction/Maintenance)	ES-SCLC patients in the first-line setting	PFS, OS, ORR, Safety	[75]

Abbreviations: extensive-stage small-cell lung cancer, ES-SCLC; overall survival, OS; progression-free survival, PFS; overall response rate, ORR: duration of response, DOR.

## Data Availability

The data either resides within this article itself or can be obtained from the authors upon making a reasonable request.

## Abbreviations

The following abbreviations are used in this manuscript:

**Abbreviation**	**Full Term**
LS-SCLC	Limited-stage small-cell lung cancer
SCLC	Small-cell lung cancer
ES-SCLC	Extensive-stage small-cell lung cancer
PCI	Prophylactic cranial irradiation
cCRT	Concurrent chemoradiotherapy
ICIs	Immune checkpoint inhibitors
NCCN	National Comprehensive Cancer Network
ESMO	European Society for Medical Oncology
BiTEs	Bi-specific T-cell engagers
IHC	Immunohistochemical
NSCLC	Non-small-cell lung cancer
TTF-1	Thyroid transcription factor-1
RB1	Retinoblastoma 1
TP53	Tumor suppressor protein 53
MYC	Myelocytomatosis
FHIT	Fragile Histidine Triad Diadenosine Triphosphatase
ASCL1	Achaete-scute homolog 1
NEUROD	Neurogenic Differentiation Factor 1
POU2F3	POU Class 2 Homeobox 3
HPF	High-pass filter
MRI	Magnetic resonance imaging
CT	Contrast-enhanced computed tomography
PET-CT	Positron emission tomography–contrast-enhanced computed tomography
EP	Platinum agent and etoposide
OS	Overall survival
PFS	Progression-free survival
TRT	Thoracic radiotherapy
AEs	Adverse events
ORR	Overall response rate
DOR	Duration of response
